# Two-Species Migration and Clustering in Two-Dimensional Domains

**DOI:** 10.1007/s11538-017-0331-0

**Published:** 2017-08-18

**Authors:** Lawrence Kurowski, Andrew L. Krause, Hanako Mizuguchi, Peter Grindrod, Robert A. Van Gorder

**Affiliations:** Mathematical Institute, University of Oxford, Andrew Wiles Building, Radcliffe Observatory Quarter, Woodstock Road, Oxford, OX2 6GG UK

**Keywords:** Fitness-dependent dispersal, Colony formation, Reaction–diffusion–advection system

## Abstract

We extend two-species models of individual aggregation or clustering to two-dimensional spatial domains, allowing for more realistic movement of the populations compared with one spatial dimension. We assume that the domain is bounded and that there is no flux into or out of the domain. The motion of the species is along fitness gradients which allow the species to seek out a resource. In the case of competition, species which exploit the resource alone will disperse while avoiding one another. In the case where one of the species is a predator or generalist predator which exploits the other species, that species will tend to move toward the prey species, while the prey will tend to avoid the predator. We focus on three primary types of interspecies interactions: competition, generalist predator–prey, and predator–prey. We discuss the existence and stability of uniform steady states. While transient behaviors including clustering and colony formation occur, our stability results and numerical evidence lead us to believe that the long-time behavior of these models is dominated by spatially homogeneous steady states when the spatial domain is convex. Motivated by this, we investigate heterogeneous resources and hazards and demonstrate how the advective dispersal of species in these environments leads to asymptotic steady states that retain spatial aggregation or clustering in regions of resource abundance and away from hazards or regions or resource scarcity.

## Introduction

We are concerned with the dispersal and clustering of populations of two-species interacting in two-dimensional spatial domains. Previously, Grindrod proposed a model controlling the dispersal of individuals in single and multispecies communities (Grindrod 1988, 1991). He took the flux of individuals to depend directly upon local population densities without requiring intermediate attractants or repellents. In such models, each individual reacts directly to other individuals in its own locality and moves so as to increase its likelihood of survival. The dynamics of such models in one spatial domain were considered in Grindrod (1988). Clustering and other interesting dynamics were observed on the finite one-dimensional spatial domain for short time scales. The evolution of conditional dispersal strategies in the context of competition between two species that are ecologically identical except in their dispersal mechanisms was considered in Chen et al. (2008). The spatial dependence of such dynamics was then considered in Hambrock and Lou (2009), when a spatially heterogeneous domain was considered.

The concept of fitness-dependent dispersal was discussed further in Armsworth and Roughgarden (2008), where models of clines with fitness-dependent dispersal were considered. This built on theoretical work of Grindrod (1988), as well as more recent work in the area of fitness-dependent dispersal (Abrams 2007; Abrams et al. 2007; Amarasekare 2007; Armsworth and Roughgarden 2005a, b; Hadany et al. 2004; Ruxton and Rohani 1998). It has been suggested that time spent moving along resource gradients (more generally, searching out resources) can limit mobility of species, relative to if the species were to simply disperse randomly (Rowell 2009). Rowell (2009) also suggests that this dispersion along resource gradients can also result in an increased likelihood of colony formation. For more on resource tracking, see Flaxman and Lou (2009).

Shigesada et al. (1979) introduced cross-diffusion models in order to model dispersal with movement up resource gradients or to avoid crowding. This involved the inclusion of logistic type terms under the diffusion operator, such as $\Delta u_i (\alpha + \beta u_i + \gamma u_j)$ and so on. A discussion on the roles that diffusion, self-diffusion, and cross-diffusion play in such models was provided in Lou and Ni (1996), and pattern formation for such models on homogeneous spatial domains was also considered. Similarly, Gambino et al. (2013) show that cross-diffusion in 2D spatial domains can lead to pattern formation. Lou and Winkler (2015) give existence and boundedness results for the Shigesada–Kawasaki–Teramoto cross-diffusion model for two competing species in the case where both species have the same diffusion coefficients and the space dimension is less than or equal to three.

Cantrell et al. (2013) discuss random dispersal versus fitness-dependent dispersal. In this and related studies, explicit fitness dependence is included in the dispersal terms (often, it is also prescribed as a modeling assumption). This is in contrast to the models of Grindrod (1988, 1991) for which the optimal direction in which the populations advect is solved for using additional equations which depend on the fecundity gradient. In this way, one can view the Grindrod model as spatially non-local in the reaction equations (one must solve for the advection direction in terms of the fecundity, which depends on the unknown population densities), while in standard fitness-dependent dispersal this additional spatial requirement is not present.

As the random dispersal rate approaches zero in these kinds of models, the equilibrium distribution of the population matches the resource density wherever the species is present. The population in occupied sections of the habitat will have equal fitness, and there will be no net movement of individuals. Such a spatial distribution is referred to as the ‘ideal free distribution’ for the population. In contrast, fitness-dependent dispersal can lead to a partitioning based on the fitness of the various individuals. Cosner (2005) proposed a dynamic model for the ideal free distribution as a partial differential equation. The well-posedness of this model was later discussed by Cosner and Winkler (2014). Cantrell et al. (2008) discuss the approximation of the ideal free distribution via reaction–diffusion–advection equations. Lou et al. (2014) consider two-species competing models with fitness-dependent dispersal in the ideal free distribution framework. Li (2015) consider two-species competition models with fitness-dependent dispersal on non-convex bounded domains under the ideal free distribution.

Nasreddine (2012) investigated the local existence and uniqueness of the one species version of the Grindrod (1988) model. Their results were extended to global existence results for two-dimensional spatial domains in Nasreddine (2014). We are aware of no corresponding results for multiple species models of the form discussed in Grindrod (1988, 1991).

In the present paper, we extend models of individual aggregation or clustering in one-dimensional domains (Grindrod 1988, 1991) to two-dimensional convex spatial domains, in order to study the possible dynamics. This allows for the two populations to move in more realistic ways than would be possible if only one spatial dimension were considered. For instance, the prey in predator–prey dynamics in a one-dimensional domain cannot escape from a predator in a finite interval, while in two spatial dimensions, the prey can attempt to evade the predator.

The remainder of the paper is organized as follows. In Sect 2, we formulate the two-species model in two spatial dimensions. We assume that the domain is finite and that there is no population flux into or out of the domain. The motion of the species is along fitness gradients which allow the species to seek out a resource. In the case of competition, species which exploit the resource alone will disperse while avoiding one another. In the case where one of the species is a predator which exploits the other species, that species will tend to move toward the prey species, while the prey will tend to avoid the predator. In Sect. 3, we consider the existence and stability of uniform steady states for spatially homogeneous resources, showing that the long-time dynamics are due primarily to the reaction kinetics. We also provide numerical evidence that these spatially homogeneous states appear to be generic behaviors of the model. We also demonstrate that on non-convex domains either homogeneous or heterogeneous steady states may be stable (depending on the form of the domain). In Sect. 4, we investigate the effects of heterogeneous resources, as well as the introduction of spatial hazards in the model. We see that the individual preferences of each species strongly dictate the dynamics, as does the heterogeneity of the domain. Discussions and concluding remarks are given in Sect.  5.

## Two-Species Model on a Two-Dimensional Spatial Domain

The dimensional form of Grindrod’s two-species competition model (Grindrod 1988, 1991) with generalized kinetics is

1 $$\begin{aligned} \frac{\partial \hat{u}_{1}}{\partial t}= & {} \hat{\delta }_{1} \Delta \hat{u}_{1}-\mu _1 \nabla \cdot (\hat{u}_{1}\hat{\mathbf {w}}_{1})+\hat{r}_1\hat{u}_{1}\hat{E}_{1}(\mathbf {x},\hat{u}_{1}, \hat{u}_{2}), \end{aligned}$$

2 $$\begin{aligned} \frac{\partial \hat{u}_{2}}{\partial t}= & {} \hat{\delta }_{2} \Delta \hat{u}_{2}-\mu _2 \nabla \cdot (\hat{u}_{2}\hat{\mathbf {w}}_{2})+\hat{r}_2\hat{u}_{2}\hat{E}_{2}(\mathbf {x},\hat{u}_{1}, \hat{u}_{2}), \end{aligned}$$

3 $$\begin{aligned} -\hat{\varepsilon }_{1}\Delta \hat{\mathbf {w}}_{1} + \hat{\mathbf {w}}_{1}= & {} \nabla \hat{E}_{1}(\mathbf {x},\hat{u}_{1},\hat{u}_{2}), \end{aligned}$$

4 $$\begin{aligned} -\hat{\varepsilon }_{2}\Delta \hat{\mathbf {w}}_{2} + \hat{\mathbf {w}}_{2}= & {} \nabla \hat{E}_{2}(\mathbf {x},\hat{u}_{1},\hat{u}_{2}). \end{aligned}$$

Here $\hat{u}_{i}(\mathbf {x},t)$ denotes the population density of species *i* at $\mathbf {x} \in \mathbb {R}^2$, $t>0$, $\hat{\mathbf {w}}_i$ is the average velocity of those individuals of population *i* dispersing, and $\hat{E}_i$ is the projected net rate of reproduction (fecundity) with $\hat{\delta }_{i} \in (0,1)$, $\hat{r}_i, \mu _i, \hat{\varepsilon }_{i} \ge 0$.

We make the model non-dimensional in such a way that the advection coefficients, $\mu _i$, are unity. Dropping the hats to obtain non-dimensional variables, we can then write Eq. (1) as

5 $$\begin{aligned} \frac{\partial u_{i}}{\partial t}=r_{i}u_{i}E_{i}(\mathbf {x},u_{1}, u_{2}) + \delta _{i} \Delta u_{i} -\nabla \cdot (u_{i}\mathbf {w}_{i}), \end{aligned}$$

6 $$\begin{aligned} -\varepsilon _{i}\Delta \mathbf {w}_{i} + \mathbf {w}_{i} = \nabla E_{i}(\mathbf {x},u_{1},u_{2}) , \end{aligned}$$

where $u_{i} \ge 0$, $\varepsilon _{i} \ge 0$, $r_i \ge 0$ and $\delta _i \in (0,1)$. Note that $r_{1}$ and $r_{2}$ can be considered as the efficiency of using fecundity or the efficiency of using the information about the abundance of a resource, or the existence of a hazard. Note that $-\varepsilon _{i}\Delta \mathbf {w}_{i}$ is the effective local average for smoothing which gives some notion of imprecise knowledge of fitness gradients and helps regularize the advective dynamics. Equations (5) and (6) are the most general formulation, and the $\mathbf {w}_i$ may not be irrotational in general for $\varepsilon > 0$.

In order to simplify the solution procedure, we define $\phi _i$ so that $\mathbf {w}_{i}=\nabla \phi _{i}$, where $\phi _i$ is a velocity potential of $\mathbf {w}_i$. We are forcing $\mathbf {w}_i$ to be the gradient of a potential, $\phi _i$, and we are ruling out the existence of any non-trivial rotational part. While this will invariably rule out some dynamics, this simplification of the model will be sufficient in order to observe the types of behaviors we are interested in studying. We obtain

7 $$\begin{aligned} -\varepsilon _{i}\Delta \phi _{i} + \phi _{i}= E_{i}(\mathbf {x},u_{1},u_{2}). \end{aligned}$$

We also rewrite Eq. (5) by using the $\phi _i$, obtaining

8 $$\begin{aligned} \frac{\partial u_{i}}{\partial t}=r_{i}u_{i}E_{i}(\mathbf {x},u_{1}, u_{2}) + \delta _{i} \Delta u_{i} -\nabla \cdot (u_{i}\nabla \phi _{i}). \end{aligned}$$

Solutions of (7) and (8) reflect solutions of (5) and (6) under the additional assumption of $\mathbf {w}_i$ being irrotational.

The results of Theorem 2.6 of Nasreddine (2012) imply convergence of solutions uniformly in *t* for $\varepsilon \rightarrow 0$ of the one species model in one dimension with spatially homogeneous logistic kinetics. Assuming that the well-posedness results from (Nasreddine 2014) can be extended to the two-species case given by Eq. (1), along with some technicalities about the compactness of solutions, we expect this simplification to be asymptotically justified for $\varepsilon \ll 1$, since $\varepsilon =0$ implies $\phi _i = E_i$ and hence $\mathbf {w}_i = \nabla E_i$.

In two-dimensions, the prey can move around the domain in order to evade the predator, while in one dimension the prey cannot ‘pass through’ the predator and hence will be caught. Microscale effects are smoothed over in the PDE formulation as it describes population distributions. However, possible directions of movement are not simply along parallel lines (which would be equivalent to the one-dimensional case). While rotational motion due to movement toward a resource is neglected for this study, the dynamics do allow for dispersion and advection. These need not be in a uniform direction. For instance, two distinct groups of prey in different regions may move in different directions to evade predators (so, the direction of movement is not along parallel lines). We also observe colony formation and dispersion of groups as seen in the one-dimensional model (Grindrod 1988). Hence, a wide variety of dynamics are observed even without pure rotation within the advection part of the equation. Note that the Laplacians in Eq. (8) still permit rotational motion as part of the dispersion process (as those Laplacians are permitted to have a non-trivial rotational part), this is just separate from the motion toward a resource or toward/away from another population.

We extend the one-dimensional spatially homogeneous setting in Grindrod (1988, 1991) by setting $E_{1}$ and $E_{2}$ as

9 $$\begin{aligned} \begin{aligned} E_{1}(\mathbf {x},u_{1},u_{2})&= A_{1}(\mathbf {x})-a_{1}u_{1}-b_{1}u_{2}-d_{1}(\mathbf {x}),\\ E_{2}(\mathbf {x},u_{1},u_{2})&= A_{2}(\mathbf {x})-a_{2}u_{1}-b_{2}u_{2}-d_{2}(\mathbf {x}). \end{aligned}\end{aligned}$$

The fecundity terms $E_i$ depend on the following parameters. The nonnegative parameters $A_{1}$ and $A_{2}$ express how the populations $u_1$ and $u_2$ exploit a spatially distributed resource. If $A_i$ is large, the populations have a greater chance to survive. The parameters $a_{1}$ and $b_{2}$ are intraspecies interaction terms. These parameters are positive, to signify that members of the same species will tend to spread out in order to more effectively exploit a common resource. The parameters $a_{2}$ and $b_{1}$ are interspecies interaction terms. Positive values indicate that the species repel one another, while negative values imply cooperation or attraction between species. In the case of competition dynamics, the two populations should repel one another, in order to model competition for a common resource. On the other hand, when one parameter is positive and one is negative, the species for which the parameter is negative can be viewed as a predator which is attracted to the other species, and that other species acts as the prey (and is repelled by the predator). Finally the positive parameters $d_i$ represent hazards or dangerous regions where the populations locally die if $d_i(\mathbf {x}) \ge A_i(\mathbf {x})$. We incorporate these hazard effects into the fecundity terms to represent hostile regions that the species’ can actively avoid via advective dispersal. One could compare these effects with hazard functions introduced into the equations for $u_i$, but not in $E_i$ and hence not in the equations for $\phi _i$. These would represent ‘invisible’ hostile regions, but for brevity we do not consider this here.

We set the initial condition as

10 $$\begin{aligned} u_{1}(\mathbf {x},0)=U_{1}(\mathbf {x})\quad \text {and} \quad u_{2}(\mathbf {x},0)=U_{2}(\mathbf {x}), \end{aligned}$$

where $U_{1}(\mathbf {x}) \ge 0$ and $U_{2}(\mathbf {x})\ge 0$ denote the initial population distributions. We consider Neumann boundary conditions so that there can be no flux of the populations either into or out of the domain $\Omega$. Let $\mathbf {n}$ be the outer normal to the domain at $\mathbf {x} \in \partial \Omega$. We have the Neumann conditions

11 $$\begin{aligned} \mathbf {n} \cdot \nabla u_{1} = \mathbf {0},~\mathbf {n} \cdot \nabla u_{2} = \mathbf {0},~ \mathbf {n} \cdot \mathbf {w}_{1}=\mathbf {n} \cdot \nabla \phi _{1}=\mathbf {0},~\mathbf {n} \cdot \mathbf {w}_{2}=\mathbf {n} \cdot \nabla \phi _{2}=\mathbf {0}, \end{aligned}$$

for $\mathbf {x} \in \partial \Omega$, $t\ge 0$. From these conditions, note that we have that

12 $$\begin{aligned} \mathbf {n}\cdot \left( \delta _i \nabla u_i - u_i \mathbf {w}_i \right) = 0, \quad i = 1,2, \end{aligned}$$

which is exactly the expected no-flux condition. Therefore, for this system, Neumann conditions on each of the four partial differential equations imply no-flux conditions hold on the two reaction–diffusion–advection equations. When solving the system numerically, we shall always implement the Neumann conditions (11).

## Spatially Homogeneous Domains

We now demonstrate the existence and sketch some results about the stability of uniform steady states for the problem (5) when the fecundity terms $E_i$ do not depend on $\mathbf {x}$. In this setting, we also neglect hazards and so set $d_i=0$, as any constant $d_i$ can just be added to the resource constants $A_i$. Grindrod (1988) had previously derived a stability criteria for dynamics of two-species in one spatial dimension. Throughout this section, we shall always assume $A_1, a_1, b_1, b_2 >0$, while $A_2 \ge 0$ and $a_2 \in \mathbb {R}$. Population $u_1$ will avoid $u_2$ ($b_1>0$) and will disperse from members of its own species ($a_1>0$), while utilizing a resource measured by $A_1>0$. Population $u_2$ will disperse from members of its own species ($b_2>0$). However, we have three possibilities for the other parameters. If we have competition dynamics, where both species must share a common resource, we have $A_2>0$ and $a_2>0$. If we have predator–prey dynamics, where population $u_2$ serves as the predator and is attracted to $u_1$ (the prey in this scenario), then we must have $A_2=0$ and $a_2 <0$. In the case where $u_2$ is a generalist predator which preys on $u_1$ yet also makes use of a shared resource, we have $A_2 >0$ and $a_2<0$. In this case, type of interaction is often referred to as intraguild predation, and it is often modeled by having three equations for the intraguild predator, intraguild prey, and resource (Ryan and Cantrell 2015). We note that our assumptions on $b_2$ and $A_2$ imply that our analysis does not extend to the classical Lotka–Volterra model (Volterra 1931) which has the form $A_2 < 0$ and $b_2=0$, but the results in this case are broadly similar, although typically less stable.

### Feasibility of Uniform Steady States

Under the assumption of uniform steady states, the model equations reduce to

13 $$\begin{aligned} \begin{aligned} 0&=r_{1}u_{1}^{*}\left( A_{1}-a_{1}u_{1}^{*}-b_{1}u_{2}^{*}\right) ,\\ 0&=r_{2}u_{2}^{*}\left( A_{2}-b_{2}u_{2}^{*}-a_{2}u_{1}^{*}\right) ,\\ \varphi _{1}^{*}&=\left( A_{1}-a_{1}u_{1}^{*}-b_{1}u_{2}^{*}\right) ,\\ \varphi _{2}^{*}&=\left( A_{2}-a_{2}u_{1}^{*}-b_{2}u_{2}^{*}\right) . \end{aligned} \end{aligned}$$

We obtain four possible uniform steady states from (13). These are

14 $$\begin{aligned} \left( u_{1}^{*},u_{2}^{*},\varphi _{1}^{*},\varphi _{2}^{*}\right) =(0,0,A_{1},A_{2}), \end{aligned}$$

15 $$\begin{aligned} \left( u_{1}^{*},u_{2}^{*},\varphi _{1}^{*},\varphi _{2}^{*}\right) =\left( 0,\frac{A_{2}}{b_{2}},A_{1}-b_{1}\frac{A_{2}}{b_{2} },0\right) , \end{aligned}$$

16 $$\begin{aligned} \left( u_{1}^{*},u_{2}^{*},\varphi _{1}^{*},\varphi _{2}^{*}\right) =\left( \frac{A_{1}}{a_{1}},0,0,A_{2}-a_{2}\frac{A_{1}}{a_{1}}\right) , \end{aligned}$$

17 $$\begin{aligned} \left( u_{1}^{*},u_{2}^{*},\varphi _{1}^{*},\varphi _{2}^{*}\right) = \left( \frac{A_{1}b_{2}-A_{2}b_{1}}{a_{1}b_{2}-a_{2}b_{1}},\frac{A_{2}a_{1}-A_{1}a_{2}}{a_{1}b_{2}-a_{2}b_{1}},0,0\right) , \end{aligned}$$

for $a_{1} b_{2} \ne a_{2}b_{1}$. The uniform steady state (14) corresponds to mutual extinction of the populations. The steady states (15) and (16) correspond to extinction of populations $u_1$ and $u_2$, respectively. Finally, the steady state (17) corresponds to the case where both populations are able to survive. The values the steady state populations take will depend on the resource availability (e.g., land fertility) divided by intraspecies competition minus interspecies species interaction, which considers the preference toward the other species.

The steady states (14) and (16) always exist. The steady state (15) exists only if $A_2 >0$ [otherwise, it reduces to the steady state (14) when $A_2=0$]. The existence of the steady state (17) is more complicated and is outlined in Theorem 1.

#### Theorem 1

For each of the three types of dynamics, a positive uniform steady state solution (17) exists when

(i) competition dynamics ($a_{2}>0$ and $A_{2}>0$): either
  $$\begin{aligned} \frac{b_{1}}{b_{2}}<\frac{A_{1}}{A_{2}}<\frac{a_{1}}{a_{2}} \text { or } \frac{a_{1}}{a_{2}}< \frac{A_{1}}{A_{2}} < \frac{b_{1}}{b_{2}}; \end{aligned}$$
(ii) generalist predator–prey dynamics: for
  $$\begin{aligned} \frac{b_{1}}{b_{2}} < \frac{A_{1}}{A_{2}}; \end{aligned}$$
(iii) and always for predator–prey dynamics.

#### Proof

(i) For competition, assume $a_{2}>0$, and $A_{2}>0$. Then, from the formula for a nonzero steady state, we need $a_{1}b_{2}-a_{2}b_{1}>0$, $A_{1}b_{2}-A_{2}b_{1}>0$, $A_{2}a_{1}-A_{1}a_{2} >0$, or $a_{1}b_{2}-a_{2}b_{1}<0$, $A_{1}b_{2}-A_{2}b_{1}<0$, $A_{2}a_{1}-A_{1}a_{2} <0$. In the first case, since all parameters are positive, divide the first inequality by $b_{2}$ and $a_{2}$, the second by $b_{2}$ and $A_{2}$, and the third by $A_{2}$ and $a_{2}$. This gives

$$\begin{aligned} \frac{a_{1}}{a_{2}} -\frac{b_{1}}{b_{2}}>0, \quad \frac{A_{1}}{A_{2}}-\frac{b_{1}}{b_{2}}>0, \quad \frac{a_{1}}{a_{2}}-\frac{A_{1}}{A_{2}}>0. \end{aligned}$$

Then, manipulating the inequalities, we obtain

$$\begin{aligned} \frac{b_{1}}{b_{2}}<\frac{A_{1}}{A_{2}}<\frac{a_{1}}{a_{2}}. \end{aligned}$$

Likewise, in the second case, dividing the first inequality by $b_{2}$ and $a_{2}$, the second by $b_{2}$ and $A_{2}$, and the third by $A_{2}$ and $a_{2}$, we find

$$\begin{aligned} \frac{a_{1}}{a_{2}} -\frac{b_{1}}{b_{2}}<0, \quad \frac{A_{1}}{A_{2}}-\frac{b_{1}}{b_{2}}<0, \quad \frac{a_{1}}{a_{2}}-\frac{A_{1}}{A_{2}}<0. \end{aligned}$$

Manipulating these, we have

$$\begin{aligned} \frac{a_{1}}{a_{2}}<\frac{A_{1}}{A_{2}}<\frac{b_{1}}{b_{2}}. \end{aligned}$$

These are the only possible cases for competition.

(ii) For the generalist predator–prey dynamics, let $A_{2}>0$ and $a_{2}<0$. Then, $a_{1}b_{2}-a_{2}b_{1}>0$ and $A_{2}a_{1}-A_{1}a_{2} >0$ are always true. For the last condition, we need $A_{1}b_{2}-A_{2}b_{1} >0$. Since $A_{1}, b_{1}, A_{2}, b_{2} >0$, this holds whenever $\frac{b_{1}}{b_{2}}<\frac{A_{1}}{A_{2}}$.

(iii) For the predator–prey dynamics, $A_{2}=0$ and $a_{2}<0$. Then, the conditions $a_{1}b_{2}-a_{2}b_{1}=a_{1}b_{2}+|a_{2}|b_{1}>0$, $A_{1}b_{2}-A_{2}b_{1}= A_{1}b_{2} >0$, and $A_{2}a_{1}-A_{1}a_{2} = 0-A_{1}a_{2}=A_{1}|a_{2}|>0$ all hold, since $A_{1}, b_{1}, A_{2}, b_{2} >0$. Hence, $A_{2}=0$ and $a_{2}<0$ imply the existence of a positive predator–prey constant steady state. $\square$

### Linear Stability of Uniform Steady States

We now assume a uniform steady state plus a small perturbation,

18 $$\begin{aligned} u_{i}(\mathbf {x},t)=u_{i}^{*}+ \omega \hat{u}_{i}(\mathbf {x},t),\quad \phi _{i}(\mathbf {x},t)=\varphi _{i}^{*}+ \omega \hat{\varphi _{i}}(\mathbf {x},t),\quad i = 1,2, \end{aligned}$$

for small perturbation parameter $|\omega |\ll 1$, where $u_{1}^{*}$, $u_{2}^{*}$, $\varphi _{1}^{*}$, and $\varphi _{2}^{*}$ are constants (corresponding to uniform steady states) satisfying (13). We plug these into the model equations, to obtain, at $O(\omega )$, the equations

19 $$\begin{aligned} \frac{\partial \hat{u}_{1}}{\partial t}= & {} r_{1}\left( A_{1}\hat{u}_{1}-2a_{1}u_{1}^{*}\hat{u}_{1}-b_{1}\left( u_{2}^{*}\hat{u}_{1}+u_{1}^{*}\hat{u}_{2}\right) \right) +\delta _{1} \Delta \hat{u}_{1}\nonumber \\&-\nabla \cdot \left( u_{1}^{*}\nabla \hat{\varphi }_{1}+\varphi _{1}^{*}\nabla \hat{u}_{1}\right) ,\nonumber \\ \frac{\partial \hat{u}_{2}}{\partial t}= & {} r_{2}\left( A_{2}\hat{u}_{2}-2b_{2}u_{2}^{*}\hat{u}_{2}-a_{2}\left( u_{1}^{*}\hat{u}_{2}+u_{2}^{*}\hat{u}_{1}\right) \right) +\delta _{2} \Delta \hat{u}_{2}\nonumber \\&-\nabla \cdot \left( u_{2}^{*}\nabla \hat{\varphi }_{2}+\varphi _{2}^{*}\nabla \hat{u}_{2}\right) ,\nonumber \\ -\varepsilon _{1} \Delta \hat{\varphi }_{1} + \hat{\varphi _{1}}= & {} -a_{1}\hat{u}_{1}-b_{1}\hat{u}_{2} ,\nonumber \\ -\varepsilon _{2}\Delta \hat{\varphi _{2}} + \hat{\varphi _{2}}= & {} -a_{2}\hat{u}_{1}-b_{2}\hat{u}_{2} . \end{aligned}$$

As the equations at $O(\omega )$ are linear, linear stability of the steady states (14)–(17) can be determined by assuming the following form of the perturbations,

20 $$\begin{aligned} \hat{u}_1,\hat{u}_2,\hat{\varphi }_1,\hat{\varphi }_2 \propto \exp (\eta t +i \mathbf {k}\cdot \mathbf {x}), \end{aligned}$$

where $\mathbf {k}$ is the wavevector of the perturbation and $\eta$ is the linear growth factor; see Murray (2003). Substituting this expression into Eq. (19) we have a system of four algebraic equations. Requiring non-trivial solutions, corresponding to non-trivial Fourier expansions of the perturbations, we can set the determinant of this system to zero in order to find a dispersion relation $\eta = \eta (k^2)$ where $k = |\mathbf {k}|$ is the wavenumber. Linear stability corresponds to $\text {Re}(\eta (k^2)) < 0$ for all $k \ge 0$, although the permitted wavenumbers *k* will depend on the spectrum of the Laplacian, and hence on the domain in question.

We shall now classify the linear stability of each of the spatially uniform (homogeneous) steady states (14)–(17) on bounded domains $\Omega \in \mathbb {R}^2$ in Theorems 2–5. We do not consider the case of heterogeneous steady states, which might be stable on non-convex domains. Indeed, for non-convex domains, related reaction–diffusion systems have been shown to admit stable heterogeneous steady states (Lou and Ni 1996; Matano and Mimura 1983).

Recall that on bounded convex $\Omega \subset \mathbb {R}$, the minimal eigenvalue of the Laplacian with Neumann boundary conditions is zero, meaning that the minimal wavenumber is $k=0$. Let us introduce the notation $K_{\Omega }$ to denote the spectrum of wavenumbers for a given bounded domain $\Omega$. As we are free to pick the $\varepsilon _i$’s, we shall assume that $\varepsilon _{i} \ne k^{-2}$ for any $k\in K_{\Omega }$, otherwise this results in degeneracy. As this is a measure zero case due to $K_{\Omega }$ being discrete, we can typically ignore it. In the case where $\varepsilon _1 = \varepsilon _2 = k^2$ for some $k\in K_{\Omega }$, the only perturbation is the zero perturbation. For $\varepsilon _1 \ne \varepsilon _2$ and $k^2 = \varepsilon _i$ for one $i=1,2$, we obtain a degeneracy resulting in a nonzero perturbation.

#### Theorem 2

The extinction steady state (14) is always unstable over any bounded domain $\Omega$.

#### Proof

Consider an arbitrary perturbation of the form

21 $$\begin{aligned} (\hat{u}_1,\hat{u}_2,\hat{\varphi }_1,\hat{\varphi }_2)^T = \exp (\eta t +i \mathbf {k}\cdot \mathbf {x})(\hat{u}_{10},\hat{u}_{20},\hat{\varphi }_{10},\hat{\varphi }_{20})^T, \end{aligned}$$

where $\eta \in \mathbb {C}$, $\mathbf {k}\in \mathbb {R}^2$, and $(\hat{u}_{10},\hat{u}_{20},\hat{\varphi }_{10},\hat{\varphi }_{20})^T$ is a constant vector. The perturbation (21) satisfies the system (19) if and only if

22 $$\begin{aligned} \det \left[ \begin{matrix} r_1A_1 -\eta &{} 0 &{} 0 &{} 0 \\ - (\delta _1 -A_1)k^2 &{} &{} &{} \\ &{} &{} &{} \\ 0 &{} r_2A-2 - \eta &{} 0 &{} 0 \\ &{} - (\delta _2 - A_2)k^2 &{} &{} \\ &{} &{} &{} \\ -a_1 &{} -b_1 &{} -(1+\varepsilon _1 k^2) &{} 0 \\ &{} &{} &{} \\ -a_2 &{} b_2 &{} 0 &{} - (1+\varepsilon _2 k^2) \end{matrix} \right] = 0. \end{aligned}$$

This condition is equivalent to either of the two conditions

23 $$\begin{aligned} \eta = A_1(r_1 +k^2) - \delta _1 k^2 \end{aligned}$$

or

24 $$\begin{aligned} \eta = A_2(r_2 +k^2) - \delta _2 k^2\,. \end{aligned}$$

This means that there exist two non-trivial perturbation vectors. When condition (23) is satisfied, the perturbation is in the direction $(\hat{u}_{10},0,\hat{\varphi }_{10},\hat{\varphi }_{20})^T$; when condition (24) is satisfied, the perturbation is in the direction $(0,\hat{u}_{20},\hat{\varphi }_{10},\hat{\varphi }_{20})^T$. Now, the perturbation is linearly stable if $\text {Re}(\eta ) <0$, and this implies that each of conditions (23), (24) should be set to less than zero if we want linear stability of all perturbations to the zero steady state. This gives

25 $$\begin{aligned} A_1< \min _{k\in K_{\Omega }}\left\{ \frac{\delta _1 k^{2}}{r_1 + k^2}\right\} \quad \text {and} \quad A_2 < \min _{k\in K_{\Omega }}\left\{ \frac{\delta _2 k^{2}}{r_2 + k^2}\right\} . \end{aligned}$$

Since the rational functions of *k* are monotone increasing, the wavenumber $k=0$ corresponding to the minimal Neumann eigenvalue minimizes these rational function. Therefore, $A_1<0$ and $A_2<0$. Yet, $A_1>0$ by assumption, so linear stability is not possible. This establishes the result. $\square$

#### Theorem 3

The asymmetric steady state (15) is linearly stable (when feasible) provided that

$$\begin{aligned} A_1 < \frac{b_1}{b_2}A_2. \end{aligned}$$

#### Proof

Consider an arbitrary perturbation of the form (21). The perturbation (21) satisfies the system (19) if and only if

26 $$\begin{aligned} \det \left[ \begin{matrix} \left( A_1 - \frac{b_1}{b_2}A_2 \right) (r_1+k^2) &{} 0 &{} 0 &{} 0 \\ -\eta - \delta _1k^2 &{} &{} &{} \\ &{} &{} &{} \\ -\frac{r_2a_2A_2}{b_2} &{} - \eta - \delta _2k^2 - r_2A_2 &{} 0 &{} \frac{A_2}{b_2}k^2 \\ &{} &{} &{} \\ -a_1 &{} -b_1 &{} -(1+\varepsilon _1 k^2) &{} 0 \\ &{} &{} &{} \\ -a_2 &{} -b_2 &{} 0 &{} - (1+\varepsilon _2 k^2) \end{matrix}\right] = 0. \end{aligned}$$

This condition is equivalent to either of the two conditions

27 $$\begin{aligned} \eta = \left( A_1 - \frac{b_1}{b_2}A_2 \right) (r_1+k^2) - \delta _1 k^2 \end{aligned}$$

or

28 $$\begin{aligned} \eta = - \left( r_2 + \frac{k^2}{1+\varepsilon _2 k^2} \right) A_2 - \delta _2 k^2. \end{aligned}$$

Condition (27) implies that $\eta <0$ if and only if

29 $$\begin{aligned} A_1 < \frac{b_1}{b_2}A_2 + \min _{k\in K_{\Omega }} \left\{ \frac{\delta _1 k^{2}}{r_1 + k^2}\right\} = \frac{b_1}{b_2}A_2, \end{aligned}$$

since the right-hand side of the inequality in (29) is increasing in *k*, and therefore $k=0$ is the minimizing wavenumber.

As $A_2>0$ (by feasibility), condition (28) always gives $\eta <0$. Therefore, the result follows. $\square$

#### Theorem 4

The asymmetric steady state (16) is linearly stable (when feasible) provided that

$$\begin{aligned} A_2 < \frac{a_2}{a_1}A_1. \end{aligned}$$

#### Proof

Consider an arbitrary perturbation of the form (21). The perturbation (21) satisfies the system (19) if and only if

30 $$\begin{aligned} \det \left[ \begin{matrix} -\eta -r_1A_1-\delta _1k^2 &{} -\frac{r_1b_1A_1}{a_1} &{} \frac{A_1}{a_1}k^2 &{} 0 \\ &{} &{} &{} \\ 0 &{} \left( A_2 - \frac{a_2}{a_1}A_1\right) (r_2 + k^2) &{} 0 &{} 0 \\ &{} -\eta - \delta _2k^2 &{} &{} \\ &{} &{} &{} \\ -a_1 &{} -b_1 &{} -(1+\varepsilon _1 k^2) &{} 0 \\ &{} &{} &{} \\ -a_2 &{} -b_2 &{} 0 &{} - (1+\varepsilon _2 k^2) \end{matrix} \right] = 0. \end{aligned}$$

This condition is equivalent to either of the two conditions

31 $$\begin{aligned} \eta = \left( A_2 - \frac{a_2}{a_1}A_1 \right) (r_2+k^2) - \delta _2 k^2 \end{aligned}$$

or

32 $$\begin{aligned} \eta = - \left( r_1 + \frac{k^2}{1+\varepsilon _1 k^2} \right) A_1 - \delta _1 k^2. \end{aligned}$$

Condition (31) implies that $\eta <0$ if and only if

33 $$\begin{aligned} A_2 < \frac{a_2}{a_1}A_1 + \min _{k\in K_{\Omega }} \left\{ \frac{\delta _2 k^{2}}{r_2 + k^2}\right\} = \frac{a_2}{a_1}A_1, \end{aligned}$$

which gives the first inequality.

$A_1 >0$ by assumption, condition (32) always gives $\eta <0$. Therefore, the result follows. $\square$

#### Theorem 5

The positive steady state (17) is linearly stable (when feasible) provided that $a_1b_2 - a_2b_1 >0$.

#### Proof

Consider an arbitrary perturbation of the form (21). The perturbation (21) satisfies the system (19) if and only if

34 $$\begin{aligned} \det \left[ \begin{matrix} -\eta -r_1a_1u_1^*-\delta _1k^2 &{} -r_1b_1u_1^* &{} u_1^*k^2 &{} 0 \\ &{} &{} &{} \\ -r_2a_2u_2^*&{} -\eta -r_2b_2u_2^*- \delta _2k^2 &{} 0 &{} u_2^* k^2\\ &{} &{} &{} \\ -a_1 &{} -b_1 &{} -(1+\varepsilon _1 k^2) &{} 0 \\ &{} &{} &{} \\ -a_2 &{} -b_2 &{} 0 &{} - (1+\varepsilon _2 k^2) \end{matrix} \right] = 0. \end{aligned}$$

For simplicity of notation, we use $u_i^*$ for both positive steady states, rather than their explicit expression in terms of model parameters.

The determinant gives a quadratic polynomial in $\eta$. In the case where $k \ne \varepsilon _{i}^{-1/2}$ for either $i=1,2$, we may scale the polynomial so that it is monic, obtaining

35 $$\begin{aligned} \eta ^2 + \xi _1(k) \eta + \xi _0 (k)= 0, \end{aligned}$$

where $\xi _1(k)$ and $\xi _0(k)$ are defined as

36 $$\begin{aligned} \xi _1(k) = a_1\left( r_1 + \frac{k^2}{1+\varepsilon _1 k^2}\right) u_1^* + b_2\left( r_2 + \frac{k^2}{1+\varepsilon _2 k^2}\right) u_2^* + (\delta _1 + \delta _2)k^2 \end{aligned}$$

and

37 $$\begin{aligned} \begin{aligned} \xi _0(k)&= (a_1b_2-a_2b_1)\left( r_1r_2 + \frac{r_1k^2}{1+\varepsilon _2k^2} + \frac{r_2k^2}{1+\varepsilon _1k^2} + \frac{k^4}{(1+\varepsilon _1k^2)(1+\varepsilon _2k^2)} \right) u_1^*u_2^*\\&\quad + \,a_1\delta _2k^2 \left( r_1 + \frac{k^2}{1+\varepsilon _1 k^2}\right) u_1^* + b_2\delta _1k^2\left( r_2 + \frac{k^2}{1+\varepsilon _2 k^2}\right) u_2^* + \delta _1\delta _2 k^4. \end{aligned} \end{aligned}$$

In order to deduce the sign of the roots of (35), the condition for $\text {Re}(\eta ) <0$ is that both $\xi _1 >0$ and $\xi _0 >0$. Note that $\xi _1(0)>0$ and $\xi _1(k)$ is increasing in *k*; hence, it is always positive, so we must ensure that $\xi _0(k)$ is positive.

We see immediately that, at the minimal wave number $k=0$, $\xi _0(0) = (a_1b_2-a_2b_1)u_1^*u_2^*$. Therefore, when $a_1b_2-a_2b_1 <0$, we have $\xi _0(0)<0$. This means that (35) has a real positive root $\eta >0$; hence, the steady state is unstable. On the other hand, when $a_1b_2-a_2b_1 >0$, we have that both roots $\eta _{1,2} <0$, hence linear stability of the positive steady state. (Recall that the case $a_1b_2-a_2b_1 =0$ is ruled out by Theorem 1) This establishes the result. $\square$

For sake of comparison, we provide the following result for the non-spatial model including only the reaction kinetics.

#### Theorem 6

Consider the dynamical system

38 $$\begin{aligned} \frac{d u_{i}}{d t}=r_{i}u_{i}(A_i -a_iu_1 - b_iu_2)\,,\quad i=1,2, \end{aligned}$$

with reaction kinetics identical to those of (5). The system has four steady states, equal to the first two components of the steady states given in (14)–(17). The feasibility of each steady state is the same as given in Theorem 1, while the linear stability of each steady state is as follows.

- The steady state $\left( u_1^*,u_2^*\right) =(0,0)$ is always unstable.
- The steady state $\left( u_1^*,u_2^*\right) =\left( 0,\frac{A_2}{b_2} \right)$ is linearly stable provided $A_1 < \frac{b_1}{b_2}A_2$ and $A_2 >0$.
- The steady state $\left( u_1^*,u_2^*\right) =\left( \frac{A_1}{a_1},0 \right)$ is linearly stable provided $A_2 < \frac{a_2}{a_1}A_1$.
- The steady state $\left( u_1^*,u_2^*\right) =\left( \frac{A_{1}b_{2}-A_{2}b_{1}}{a_{1}b_{2}-a_{2}b_{1}},\frac{A_{2}a_{1}-A_{1}a_{2}}{a_{1}b_{2}-a_{2}b_{1}} \right)$ is linearly stable provided $a_1b_2 - a_2b_1 >0$.

#### Proof

The Jacobian for the system (38) evaluated at a steady state is given by

39 $$\begin{aligned} J|_{(u_1^*,u_2^*)} = \left[ \begin{matrix} r_1(A_1 -2a_1u_1^*-b_1u_2^*) &{} \quad -r_1b_1u_1^*\\ &{} \\ -r_2a_2u_2^* &{} \quad r_2(A_2 - a_2u_1^* - 2b_2u_2^*) \end{matrix}\right] . \end{aligned}$$

Using this Jacobian matrix, the proof of the four results is standard. $\square$

A comparison between Theorems 6 and 2–5 shows that advection-diffusion does not stabilize or destabilize any steady state over arbitrary perturbations, suggesting that the long-time dynamics are controlled by the reaction kinetics, with advection and diffusion controlling transient dynamics.

Note that these stability results are valid not just for two-dimensional bounded domains, but for one- or three-dimensional bounded domains, as well, as the change in spatial dimension will simply modify the elements of the spectrum $K_{\Omega }$. With zero flux conditions, the smallest eigenvalue of the Laplacian is zero; hence, the zero wavenumber will still control the stability or instability of the steady states, in general. Note also that the stability results we obtain are distinct from those of the one-dimensional two-species model in Grindrod (1988), as those results corresponded to the $r_1,r_2\rightarrow 0$ limit, hence the present results are more general.

We should also remark that the stability results of Grindrod (1988) were given in terms of fixed wavenumbers, rather than for general perturbations. A general perturbation will involve a superposition of modes over all of $K_{\Omega }$, rather than a fixed mode corresponding to a single wavenumber in $K_{\Omega }$. However, one can imagine situations where knowledge of the stability or instability of a uniform steady state under a very specific form of perturbation is useful. If we restrict to wave numbers above a threshold $k \ge \overline{k} >0$, then the asymmetric steady states will maintain the stability criteria given in Theorems 3 and 4, as the stability eigenvalues will not change sign for any nonnegative wavenumber. However, when $\delta _1 > r_1 A_1$ and $\delta _2 > r_2 A_2$, the zero steady state can become stable for large enough wavenumber threshold $\overline{k}$. Similarly, for large enough wavenumber threshold $\overline{k}$, the function $\xi _0(k)$ in the proof of Theorem 5 will become positive, no matter the sign of $a_1b_2 - a_2b_1$, for any $\delta _1,\delta _2 >0$. Therefore, if the perturbation contains only large wavenumbers, the spatial terms can actually stabilize the uniform positive steady states when the reaction kinetics might suggest instability.

It is presently unknown whether non-uniform steady states are necessarily unstable, although this can be shown for certain similar models in convex domains. For instance in the case without advection, Kishimoto and Weinberger (1985) show that for general competition kinetics, all non-uniform steady states are unstable on convex domains. Numerically we observe convergence only to one of the four uniform steady states (14)–(17) for all choices of kinetic parameters and initial conditions, as well as varying diffusion parameters for each species $\delta _i$. For this reason, we conjecture that stable non-uniform steady states do not exist for this model for spatially homogeneous fecundity parameters when the domain is convex. We also considered simulations for convex domains with holes, and for such non-convex domains we still observed convergence to one of the uniform steady states. Still, there may exist non-convex domains for which there are stable heterogeneous steady states, as shown for other models (Lou and Ni 1996; Matano and Mimura 1983).

### Numerical Solution Approach

Having studied the uniform steady states of the problem (5) with spatially homogeneous fecundity, we now turn our attention to transient behavior in this case, before moving on to more interesting spatially inhomogeneous resources and hazards in Sect.  4. In the numerical experiments, we take the domain to be the unit square, $\Omega =[0,1]^2$. We discretise in space using centered finite differences and apply a fourth-order Runge–Kutta scheme for the two-species model, using the Matlab function ‘ode45.’ The temporal domain can be normalized to the interval [0, 1] by selecting the maximal simulation time, $T^*$, in terms of the reaction timescale $r=r_1=r_2$ assuming all other reaction parameters are order *O*(1) (that is $T^* = T^*(r)$). In this way, $t=T^*\hat{t}$, $\delta _i = (T^*)^{-1}\hat{\delta }_i$, $\phi _i = (T^*)^{-1}\hat{\phi }_i$, $r= (T^*)^{-1}\hat{r}$. We note that $T^*$ need not be very large. From Grindrod (1988), we see that the natural time taken to arrive at a steady state solution was of order 10, and for parameter values used here, one can see stabilization even sooner for some cases (since the eigenvalues are larger in magnitude and the convergence rate from excitations to a steady state is exponential). This approach allows us to see the transient behaviors, while the long-time behaviors are rather more tame for the homogeneous parameters (as was the case in the original work in one spatial dimension Grindrod 1988, 1991). This timescale corresponds to approximately reaching steady state for $\hat{t}=1$, where we observe only uniformity in the homogeneous case, but spatial structure for the inhomogeneous case. As a check on our numerical scheme, and to consider non-convex geometries, we also simulated these equations using the finite element software COMSOL. For all such simulations, more than 50, 000 triangular elements were used.

In what follows we shall drop hats on time for convenience, with the understanding that we have scaled time appropriately so that the dynamics occur for $t\in [0,1]$. Note that the scale $T^*$ is different for each parameter combination. Since we are primarily interested in the qualitative behavior of these systems rather than quantitative analysis, we just report plots with the maximal run time already scaled to 1, as the particular value of $T^*$ in each case is not particularly interesting for our discussions. For numerical computations, the spatial domain is divided into $50 \times 50$ increments. We constrained the maximum timestep to be $10^{-2}$. Unless otherwise mentioned, we set ${\delta }_{1}, {\delta }_{2}=0.1$, $\varepsilon _{1}, \varepsilon _{2}=0.025$, $r_1=r_2=1$, and the reaction term coefficients as $a_1 = 1, a_2 = 3, b_1 = 2, b_2 = 1$ for competition dynamics and $a_1 = 1, a_2 = - 3, b_1 = 2, b_2 = 1$ for predator and generalist predator-prey dynamics. For predator–prey, we will always have $A_2 = 0$ for all $(x_1,x_2)\in \Omega$ for all numerical experiments. We will denote steady states by $u_i^*$ and by $\overline{u}_i^*=\int _\Omega u_i^*(x_1,x_2)dx_1dx_2$ we denote the steady state averaged over the domain.

### Simulations on Spatially Homogeneous Domains

We use periodic functions for the initial conditions, given by

40 $$\begin{aligned} \begin{aligned} u_{1}(x_1,x_2,0)&=\frac{1}{4} (\sin (x_1)+1)(\sin (x_2)+1),\\ u_{2}(x_1,x_2,0)&=\frac{1}{4}(\cos (2x_1)+1)(\cos (2x_2)+1). \end{aligned} \end{aligned}$$

The choice of initial conditions for the spatially homogeneous case is relatively inconsequential, and the long-time dynamics can in many cases just be predicted by the total population of each species in the domain, and the fecundity parameters.

**Fig. 1:**
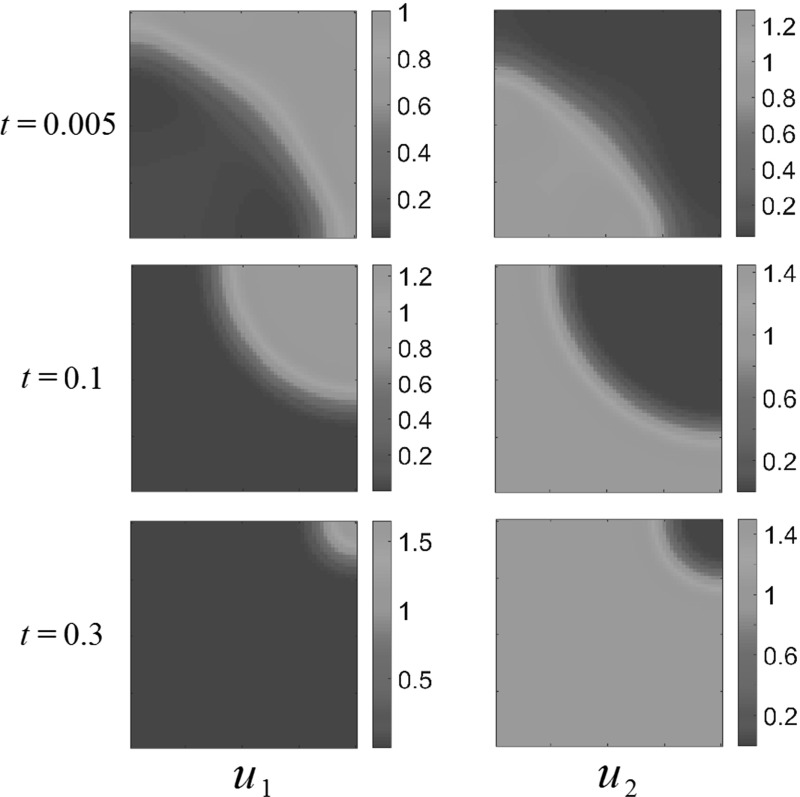
(Color figure online) Competition dynamics in a spatially homogeneous domain, with fecundity parameters $A_1=1$, $A_2=1.5$, $a_1 = 1, a_2 = 3, b_1 = 2, b_2 = 1$ using the sinusoidal initial conditions (40)

As an example of the spatially homogeneous parameters, we show competition dynamics in Fig. 1. The populations quickly separate into two distinct regions and appear to be content in their separate parts of the domain. However, on the time scale of the diffusive dispersal, $u_2$ begins to locally encroach on $u_1$ which flees into a smaller region, and is eventually driven into extinction throughout the entirety of the domain $\Omega$.

**Fig. 2:**
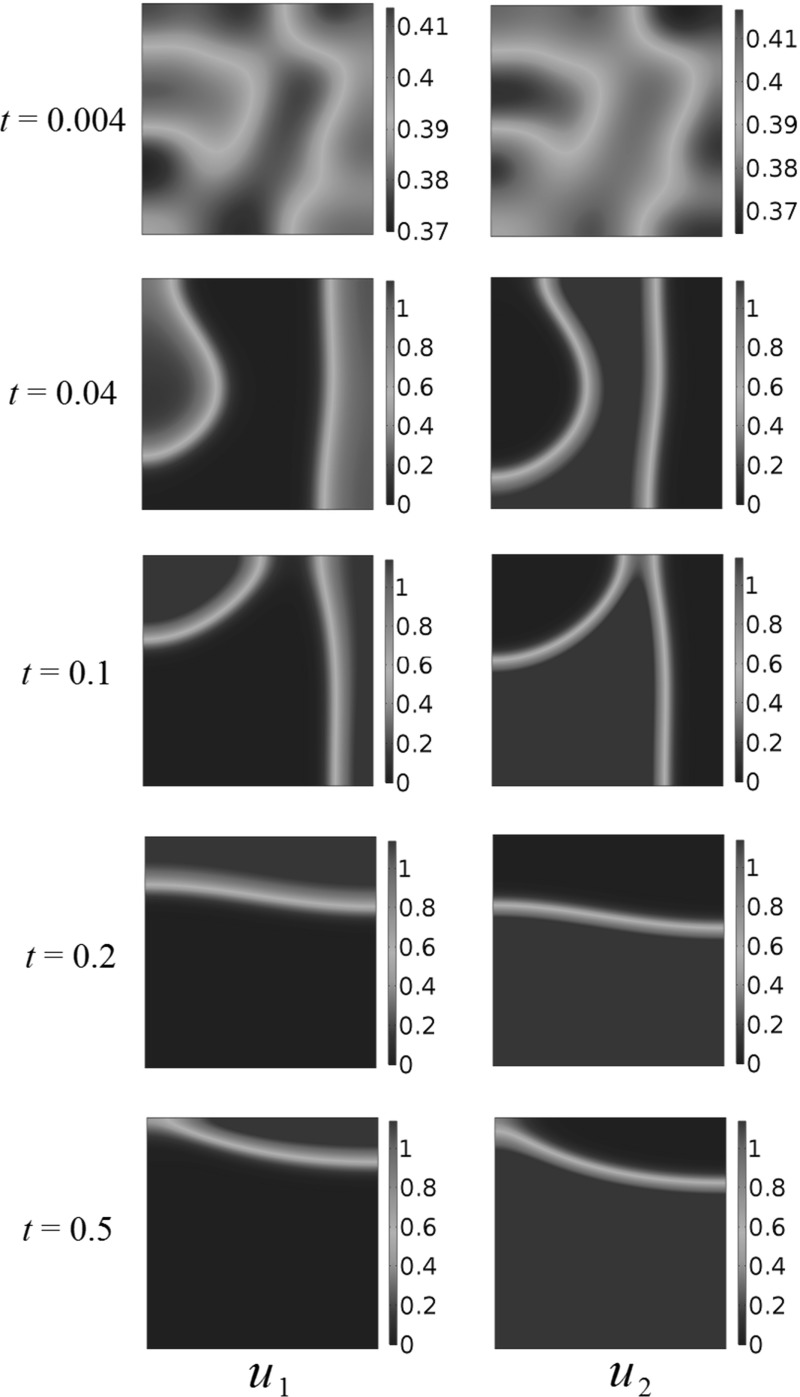
(Color figure online) Competition dynamics in a spatially homogeneous domain, with fecundity parameters $A_1=1$, $A_2=1.5$, $a_1 = 1, a_2 = 3, b_1 = 2, b_2 = 1$ using random initial conditions

As in Grindrod (1988), small random initial populations will form clusters and aggregates, but these will eventually disperse and lead to uniform steady states, which are dominated by the interaction parameters. As an example of this, consider the competition dynamics in Fig. 2. Random initial populations were generated by taking the absolute value of a normal random variate with mean 0 and variance 0.25 for each population at each finite element. These quickly aggregate with the populations clustering away from one another. Over time, the more fit population overtakes the competing species, driving it into a corner of the domain and eventually to extinction.

**Fig. 3:**
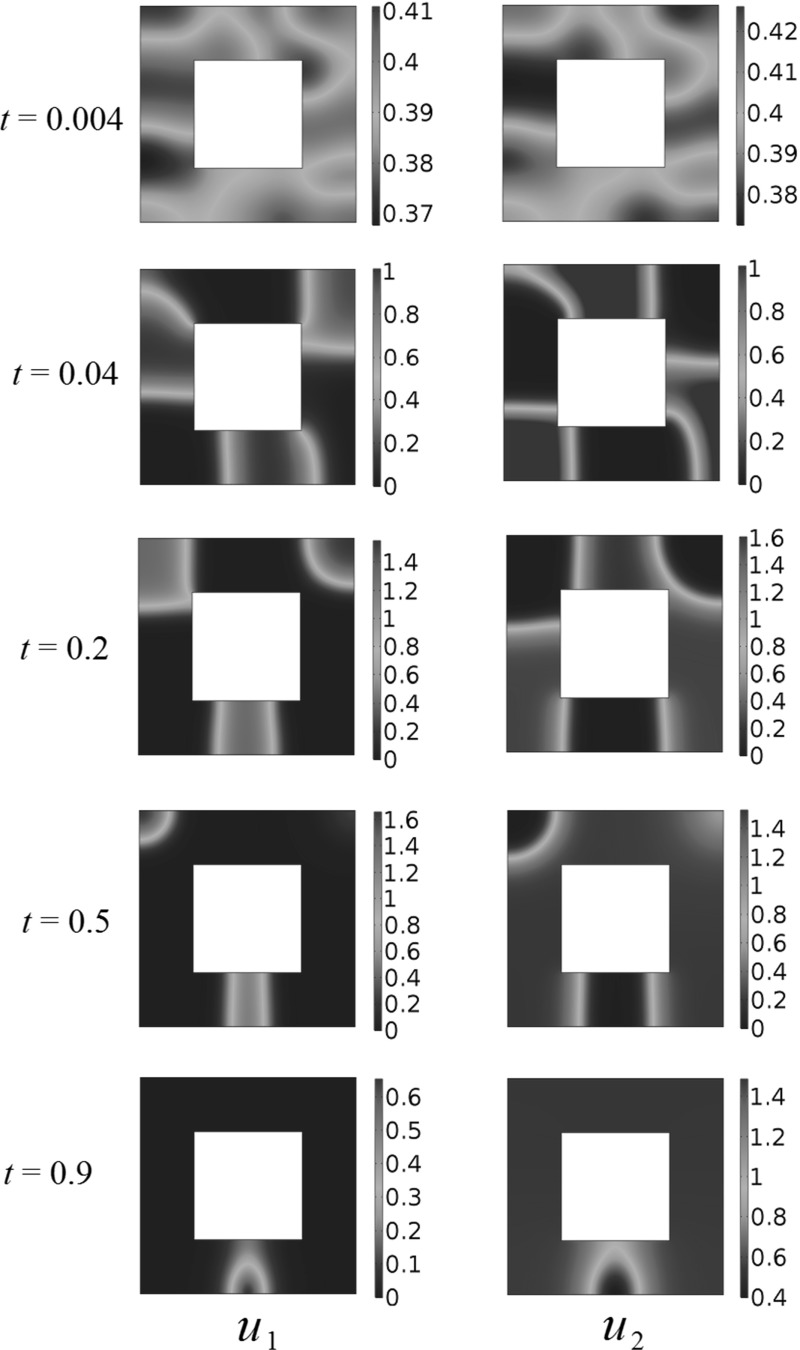
(Color figure online) Competition dynamics in a *square* domain with a hole, with fecundity parameters $A_1=1$, $A_2=1.5$, $a_1 = 1, a_2 = 3, b_1 = 2, b_2 = 1$ using random initial conditions

We now consider domains of different shapes, and in particular patterning in non-convex domains. Using the same kind of random initial data from Fig. 2, we consider a square hole of side length 0.5 in the center of the larger square domain of side length 1, and these results are displayed in Fig.  3. As in the case of the square domain, transient population clustering occurs, but eventually the population $u_1$ is driven to extinction by population $u_2$. We simulated several different realizations of initial conditions, as well as the initial conditions (40), but never observed any long-time clustering in either the square or the perforated domain.

Finally, motivated by non-uniform patterns present in non-convex domains (Matano and Mimura 1983), we consider the same competition dynamics in a ‘dumbbell’-shaped domain consisting of two squares of unit side length with a connecting rectangle between them of unit length and height 0.1. We used the following initial conditions, with $x_1=0$ denoting the midpoint of the connecting rectangle,

41 $$\begin{aligned} \begin{aligned} u_{1}(x_1,x_2,0)&=\tanh (100x_1),\\ u_{2}(x_1,x_2,0)&=\tanh (-100x_1). \end{aligned}\end{aligned}$$

**Fig. 4:**
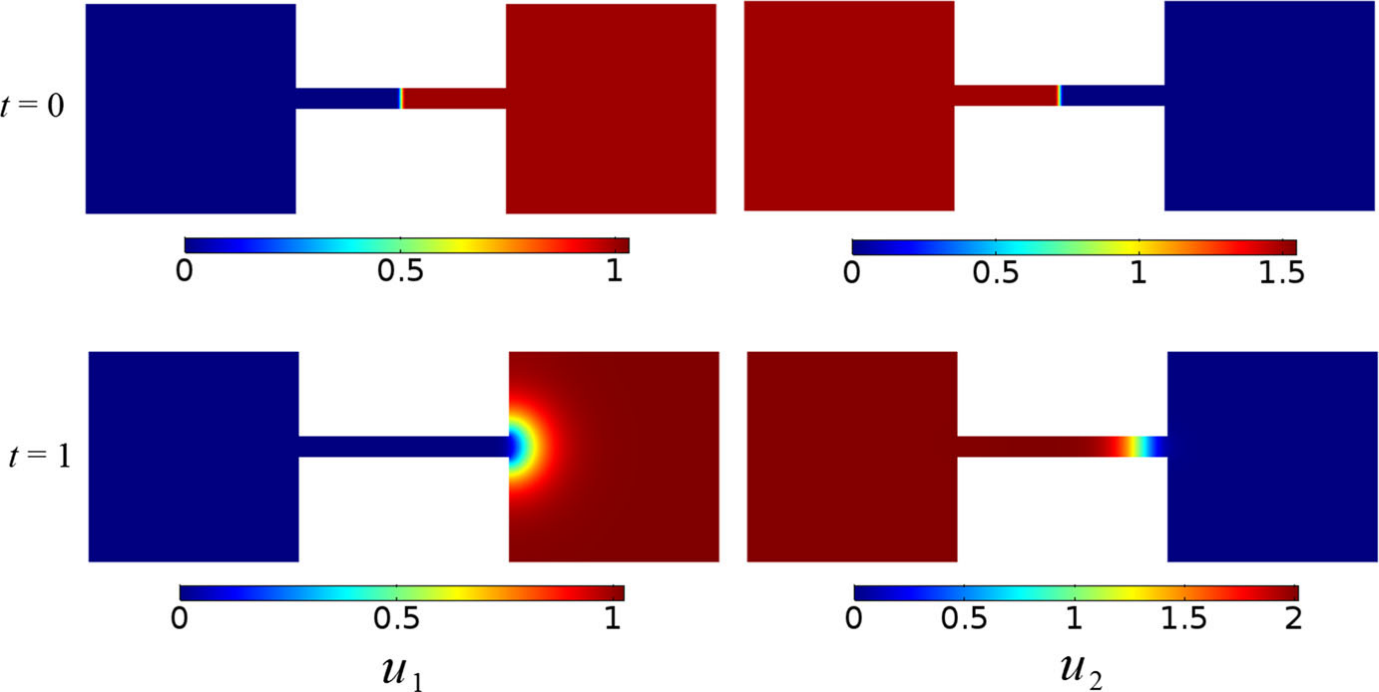
(Color figure online) Competition dynamics in a non-convex ‘dumbbell’ domain, with fecundity parameters $A_1=1$, $A_2=1.5$, $a_1 = 1, a_2 = 3, b_1 = 2, b_2 = 1$ using random initial conditions

We plot the steady state behavior of this simulation in Fig.  4. While the more fit population, $u_2$, is able to occupy most of the connecting rectangle, it is unable to invade into the right-hand side of the domain. We note that we only observed patterning in this domain if the population densities were initially very separated, as in (41). Other simulations with random initial conditions, or for some other values of the kinetic parameters, did not admit patterned populations as numerically stable steady states.

We ran many other numerical simulations which demonstrate interesting dynamics at small timescales, such as the colony formation and aggregation of populations noted in Grindrod (1988) and seen above. However, in convex domains or for approximately homogeneous initial conditions, these dynamics tended toward spatially homogeneous steady states at larger timescales, as our stability analysis implied. Therefore, the large time dynamics tending to homogeneous steady states seems robust on convex domains. We also ran simulations in cases with a non-convex domain consisting of a convex domain with a central hole in the domain, and in all of the simulations we again observed convergence to homogeneous states for sufficiently large time. No spatial patterning or non-equilibrium dynamics were observed for large time. This suggests that convergence to homogeneous steady states is fairly ubiquitous for this family of models when the domain is homogeneous, at least on convex and certain non-convex domains, and hence that the colony formation and aggregation seen at small timescales and due to a combination of diffusion and advection is indeed transient, with long-time dynamics governed by the reaction kinetics.

## Spatially Heterogeneous Domains

Since heterogeneous steady states do not appear to be stable in convex domains, we shall next turn to numerical solutions with spatially inhomogeneous parameters in the fecundity terms in order to demonstrate asymptotic clustering and aggregation mechanisms not present in the spatially homogeneous model (5). In particular, we now consider the fecundity functions $E_i$ to depend on space explicitly. We first consider spatially varying resources $A_i(\mathbf {x})$ with varying levels of spatial structure. We then consider introducing spatially dependent deaths into the functions $E_i$ to model the presence of hazards in the domain.

### Single Bump Resource

Consider the following resource functions

42 $$\begin{aligned}&A_1 (x_1,x_2) = \exp {(-(x_1-0.5)^2-(x_2-0.5)^2)}, \quad \text {for all}~~ t>0, \nonumber \\&A_2 (x_1,x_2) \nonumber \\&\quad = \left\{ \begin{array}{ll} A_1 (x_1,x_2), &{} \quad \text{ for } \text{ competition } \text{ and } \text{ generalist } \text{ predator-prey } \text{ interactions }, \\ 0, &{} \quad \text{ for } \text{ predator-prey } \text{ interactions }. \end{array} \right. \nonumber \\ \end{aligned}$$

This describes a Gaussian-shaped resource with a single peak at [0.5, 0.5], symmetric about this peak in $\Omega$. For all types of interactions, after a short transient period of motion, both populations settle onto the resource surface and interact in essentially the spatially independent ways that they do for uniform populations.

**Fig. 5:**
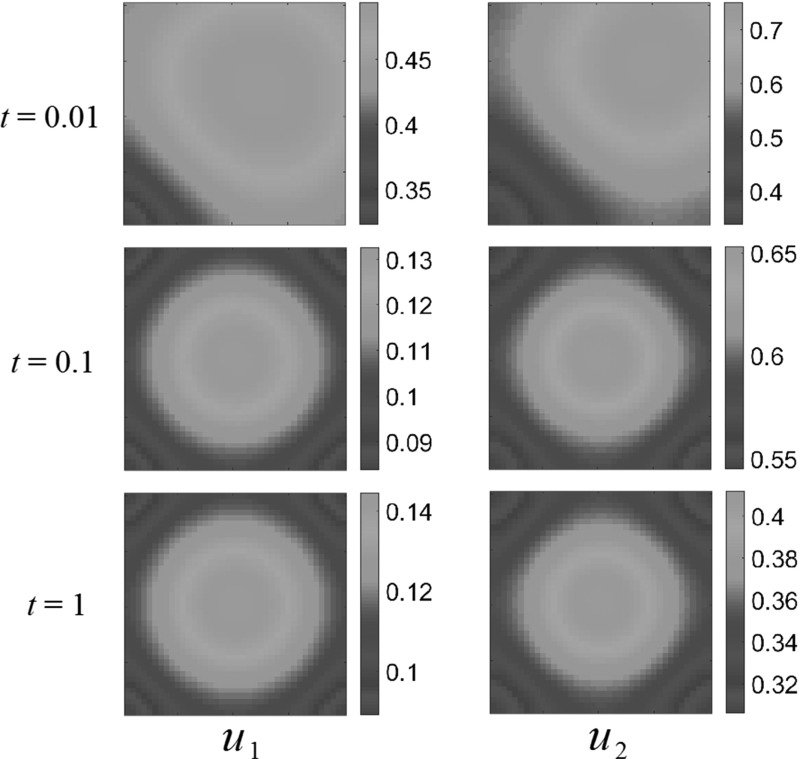
(Color figure online) Gaussian resource distribution and predator–prey dynamics. Both populations settle down to non-uniform steady states, distributed more-or-less on top of the resource surface. Reaction coefficients are $a_{1}=1$, $a_{2}=-3$, $b_{1}=2$, $b_{2}=1$

Figure 5 shows predator–prey dynamics with both species eventually settling down to a spatially heterogeneous steady state. While this steady state is not uniform, it is essentially predictable from the spatially independent kinetics $E_i$. This state appears to be spatially determined by the resource function, but quantitatively only determined by the total amount of the resource, and the parameters defining the interspecies dynamics.

To be precise, we can compare the quantitative differences between the population averages, $\overline{u}_1^*$ and $\overline{u}_2^*$, and the spatially homogeneous steady states where we average the resource functions $A_i(x_1,x_2)$ over the domain and substitute this value into Eq. 17. The steady states obtained via numerical simulation for the Gaussian resource, averaged over the domain, are in each case within $1\%$ of the values predicted for the homogeneous steady states. This was compared for several initial data and diffusion parameters $\delta _i$, and the deviations were always very small. We conjecture that this is due to the fact that the resource function is symmetric in $\Omega$ and the magnitude of $A_1$ varies only gently across the domain.

### Multiple-Bump Resource Surface

Consider the following resource distributions

43 $$\begin{aligned}&A_1 (x_1, x_2) = \frac{1}{2} + \frac{9\sin ^2{(2\pi x_1)}\sin ^2{(2\pi x_2)}}{20} ,\nonumber \\&A_2 (x_1, x_2) \nonumber \\&\quad = \left\{ \begin{array}{ll} \frac{1}{2} + \frac{9\cos ^2{(2\pi x_1)}\cos ^2{(2\pi x_2)}}{20} , &{} \quad \text{ competition } \text{ and } \text{ generalist } \text{ predator-prey } \text{ interactions }, \\ 0, &{} \quad \text{ predator-prey } \text{ interactions }. \end{array} \right. \nonumber \\ \end{aligned}$$

**Fig. 6:**
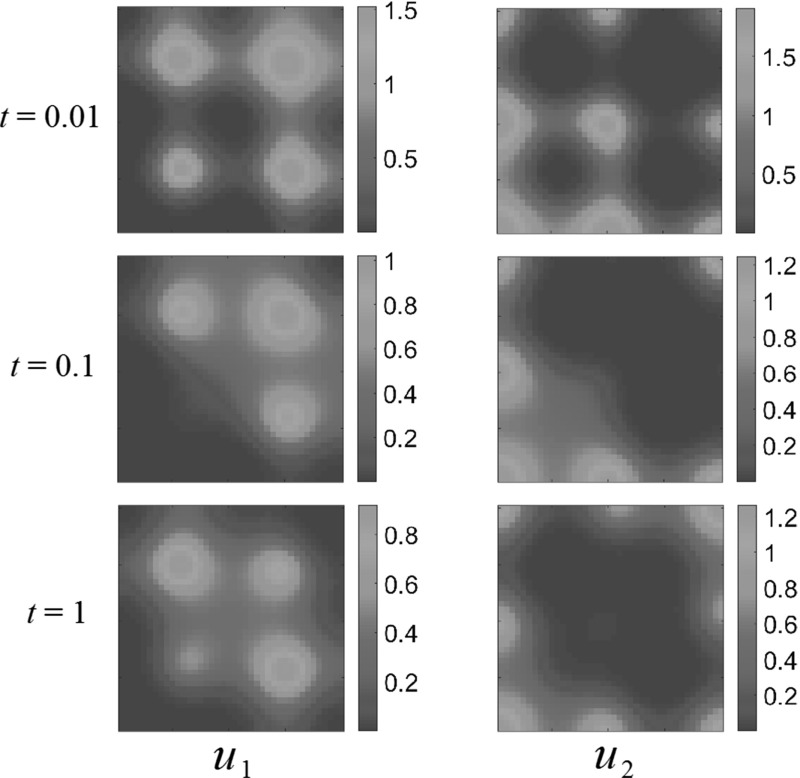
(Color figure online) Multiple-bump resource function; competition dynamics. Both populations settle down to non-uniform steady states, avoiding certain areas of the domain $\Omega$. Reaction coefficients are $a_{1}=1$, $a_{2}=3$, $b_{1}=2$, $b_{2}=1$

**Fig. 7:**
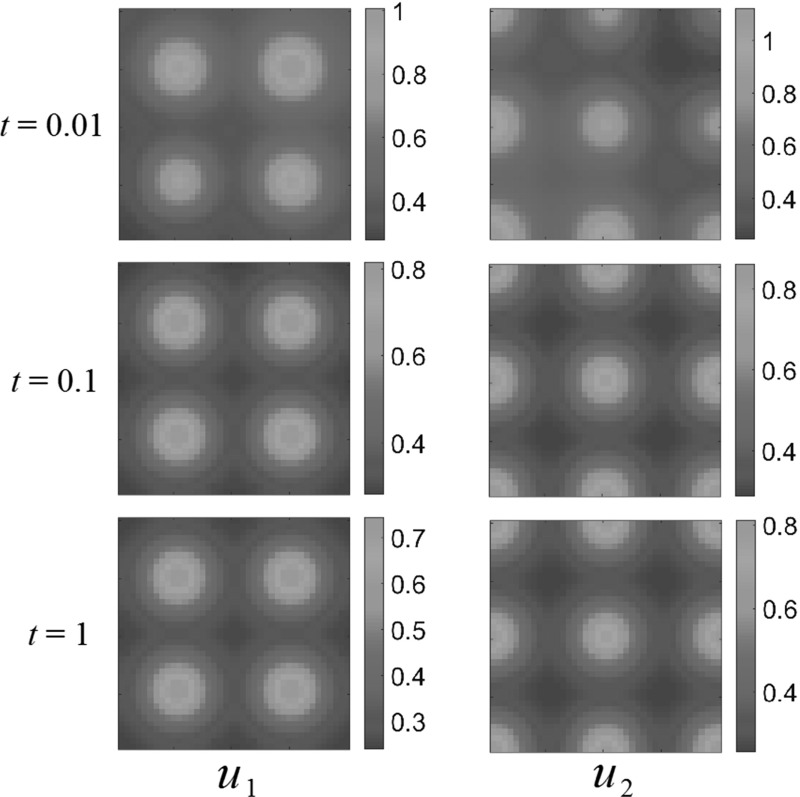
(Color figure online) Multiple-bump resource function; competition dynamics. Both populations settle down to non-uniform steady states, avoiding certain areas of the domain $\Omega$. Reaction coefficients are $a_{1}=1$, $a_{2}=0.01$, $b_{1}=0.01$, $b_{2}=1$

**Fig. 8:**
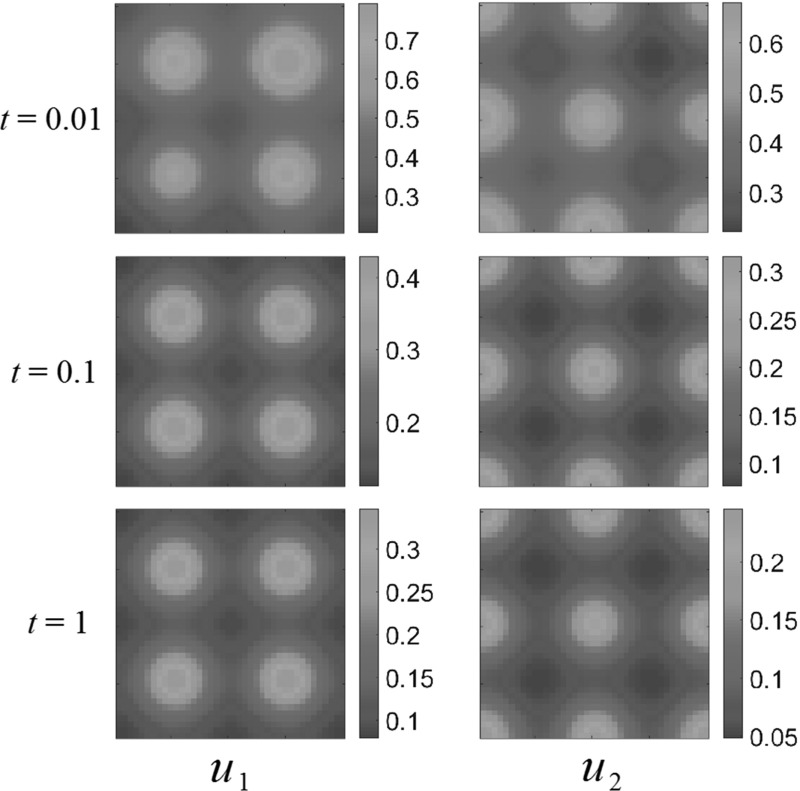
(Color figure online) Multiple-bump resource function; competition dynamics. Both populations settle down to non-uniform steady states, avoiding certain areas of the domain $\Omega$. Reaction coefficients are $a_{1}=2$, $a_{2}=1$, $b_{1}=1$, $b_{2}=3$

This describes the more spatially structured domain depicted in Figs. 6, 7 and 8. For the case of competition or generalist predator dynamics, we have varied the phase of the resource such that different parts of the domain are nutritionally dense for each species. For predator–prey dynamics, we observe the same behavior as in the previous discussion of a single bump resource: both populations accumulate where the resources are dense and are sparse elsewhere. For competition dynamics, however, we observe more spatially structured interactions between the species.

Figure 6 shows competition dynamics. $u_2$ initially expands into the domain, setting up a colony around the central resource-rich “bump” in $A_2(x,y)$. For later times, this colony is abandoned, as $u_1$ expands across the domain, driving the other species away. This suggests a spatial effect competition has on the dynamics, mediated by the dispersal in this heterogeneous environment. Note that eventually, in the steady state, both populations choose not to occupy certain resource-rich regions close to the other, competing species. There is now a noticeable effect of the initial condition that persists at steady state, and we anticipate many more possible steady states depending on the choice of initial population distributions.

To understand the competition effect in terms of the interaction parameters between the species, we now modify the reaction term coefficients, so as to reduce the competitive interaction. Let $a_1 =1, a_2 = 0.01, b_1 = 0.01, b_2 = 1$. Figure 7 shows the competition dynamics for this choice of parameters. Drastically reducing the competition coefficient did indeed allow both species to occupy all food-rich regions of $\Omega$ and again reduces the spatial structure of the populations to following the resource distributions closely.

We can also see what happens for increased intraspecies overcrowding coefficients $a_1$, and $b_2$. Let $a_1=2, a_2 = 1, b_1 = 1, b_2 = 3$. Figure 8 shows the effect this choice of parameters has on the behavior of our system. Just as with reduced competition, the advection away from competing species is weaker in this case than in the intraspecies interactions shown before, and so the steady states match the resource-rich regions of $\Omega$. This suggests that, depending on the spatial distribution of resources in the domain, we expect non-trivial interactions between the species only in certain subsets of the parameter space-namely when interspecies competition is strong.

### Half-Domain Resource

**Fig. 9:**
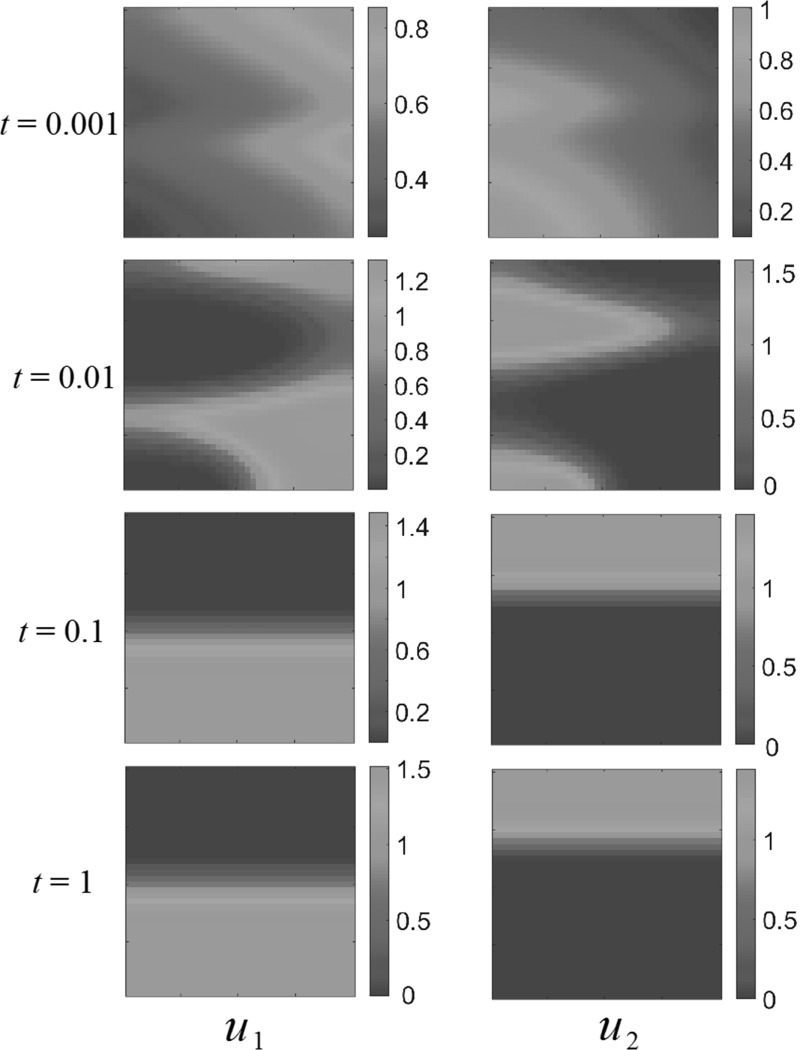
(Color figure online) Half-domain resource with competition dynamics. After transient colony formation period, populations settle down into steady states. Rather than migrating along as a group, the species form new colonies in resource-rich regions and let the original colony die out. Reaction coefficients are $a_{1}=1$, $a_{2}=3$, $b_{1}=2$, $b_{2}=1$

In many of the situations considered above, the species would generally cluster around the spatially distributed resource. We now consider their spatiotemporal interactions during a migration between two patches of resources. In Fig. 9, we plot competition dynamics under the resource distributions

44 $$\begin{aligned}&A_1 (x_1, x_2) = 1-\frac{1}{2}\tanh \left( 12\left( x_2-\frac{1}{2}\right) \right) , \nonumber \\&A_2 (x_1, x_2) \nonumber \\&\quad = \left\{ \begin{array}{ll} 1+\frac{1}{2}\tanh \left( 12\left( x_2-\frac{1}{2}\right) \right) , &{}\quad \text{ competition } \text{ and } \text{ generalist } \text{ predator-prey } \text{ interactions }, \\ 0, &{}\quad \text{ predator-prey } \text{ interactions }. \end{array} \right. \nonumber \\ \end{aligned}$$

As expected, in the long run both populations settle down to steady states in the part of $\Omega$ where there is the greatest abundance of the food they consume. In the transient dynamics, however, instead of traveling as an aggregate, the populations appear to set up new clusters in food-rich regions and let the old colony die out. This appears to be due to the advective dispersal away from the other species during the migration into the food-rich area. We can experiment to test whether this is due to the animals’ proclivity for local clustering by reducing intraspecies coefficients. If $a_1 = 0.1, a_2 = 3, b_1 = 3, b_2 = 0.1$, the populations tend to repel each other but overcrowding within each one species is subdued. We observe similar behavior to that seen in Fig. 9.

### Spatially Heterogeneous Death Rates

We now consider the effect of introducing a nonzero spatial death term $d_i \ne 0$ to the fecundity terms (9). We take the form of this to be

45 $$\begin{aligned} \begin{aligned} d_i (x_1, x_2)&= \left\{ \begin{array}{ll} 5, &{}\quad \frac{1-a}{2}<x_1, x_2< \frac{1+a}{2} \quad \text {where} \quad 0<a<1, \\ 0, &{}\quad \text {otherwise}, \\ \end{array} \right. \end{aligned} \end{aligned}$$

for $i=1, 2$. We also set $A_i(x_1,x_2) = 1$. This describes a spatially homogeneous resource with presence of a deadly square region of side length *a* in the center of the domain. Physically, this can represent a danger present in only part of the domain.

**Fig. 10:**
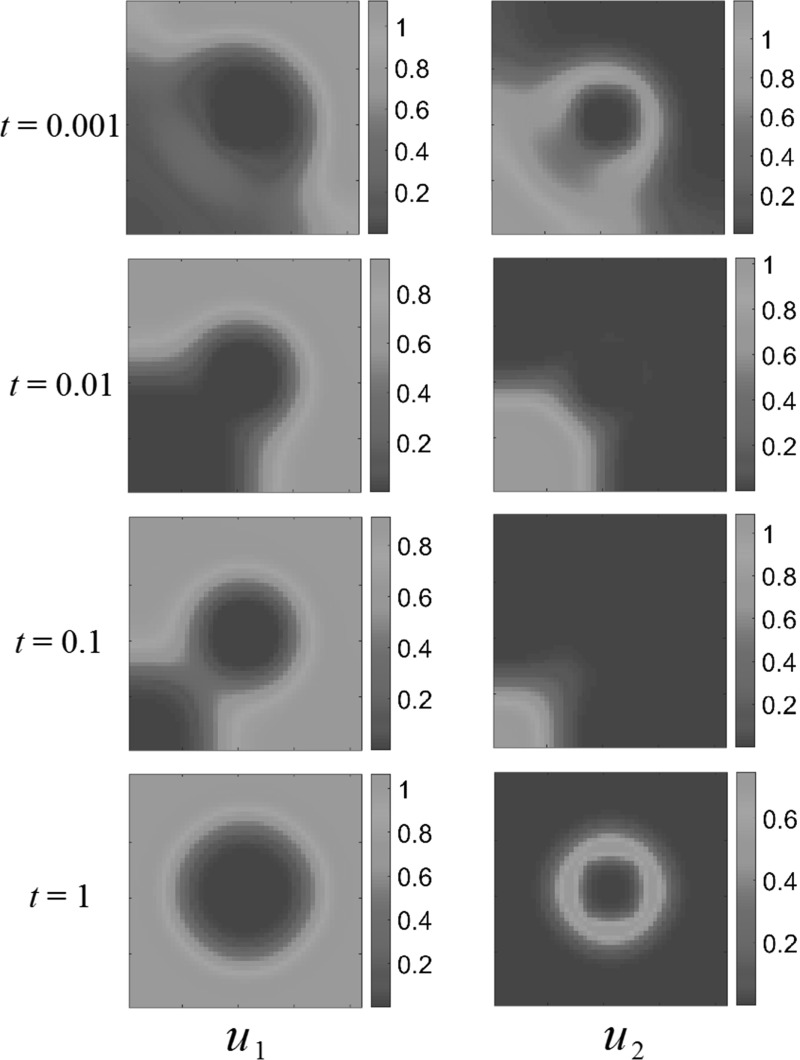
(Color figure online) Square death rates with competition dynamics. The population $u_2$ undergoes interesting transient dynamics before settling on the brim of the hazard. Reaction coefficients are $a_{1}=1$, $a_{2}=3$, $b_{1}=2$, $b_{2}=1$

Figure 10 shows competition dynamics for this model, for $a=0.2$. Note the transient behavior of $u_2$ in particular, which initially gets pushed into the bottom left corner of the domain, but instead of being driven to extinction, migrates to the middle of the square to settle down to a steady state on the brim of the hazardous region, where the generalist predator species is less prevalent. This is possible as the generalist predator species forms a circular, rather than square-like distribution around the deadly region, leaving a thin stripe of $\Omega$ capable of supporting the prey species. This circular brim is likely due to the corners of the small square inducing a smoothing effect due to the advection away from them by the predator.

**Fig. 11:**
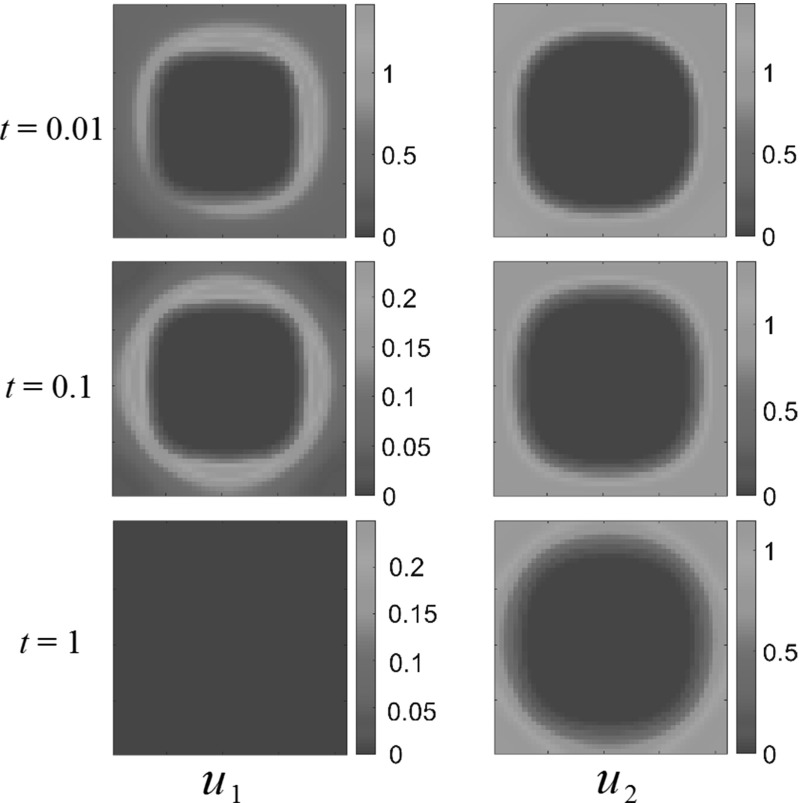
(Color figure online) Square death region with generalist predator–prey dynamics, for *square* side $a=0.5$. The prey population dies out eventually. Reaction coefficients are $a_{1}=1$, $a_{2}=-3$, $b_{1}=2$, $b_{2}=1$

We now consider generalist predator–prey dynamics. Figure 11 shows the $a=0.5$ case, where the prey species $u_1$ dies out eventually. Note how again the generalist predator population forms a circular, rather than a square, colony. This is also likely due to the advective dispersal around the corners of the hazard.

**Fig. 12:**
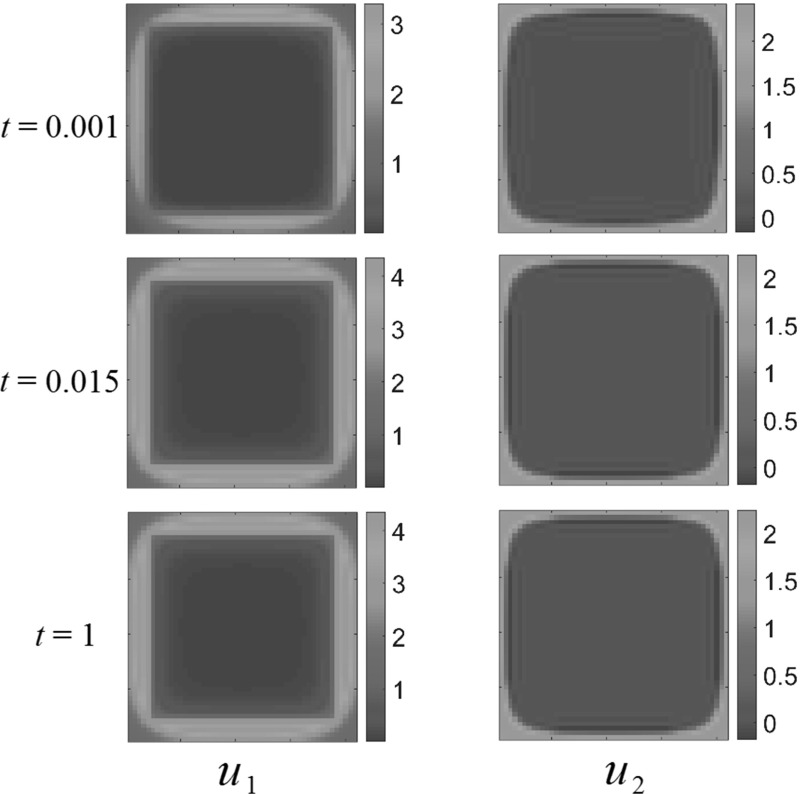
(Color figure online) Square death region with generalist predator–prey dynamics, for square side $a=0.8$. Both populations settle down to a positive steady state. Reaction coefficients are $a_{1}=1$, $a_{2}=-3$, $b_{1}=2$, $b_{2}=1$

We contrast this with the $a=0.8$ case, depicted in Fig.  12. For a larger hazard, the prey species tends to survive and settle down to a positive steady state, by aggregating along the outer rim of the domain. The generalist predator forms a circular colony, again allowing the prey to exist between the generalist predator and the hazard. Finally, as we increase the spatial size of the hazard further, we find that the prey species dies out near around $a=0.95$. There is therefore a range of values of *a* for which the prey species survives in this particular model of a hazard.

## Discussion

We have extended the model of Grindrod (1988) to study population dynamics of two competing species in two-dimensional domains. The primary benefit of including two-dimensional aggregate motion is that this permits far more realistic dispersion compared to dynamics in only one spatial dimension. Additionally, realistic heterogeneity can be directly modeled in a two-dimensional domain, allowing for the persistent aggregation of populations.

In Sect. 3, we demonstrated that three types of uniform steady states are possible: complete extinction of both species, partial extinction (one species goes extinct, the other persists), and a positive steady state (persistence of both species). We were able to give criteria for the feasibility of each in terms of the model parameters. We were then able to discuss the linear stability of these uniform steady states under small perturbations obeying Neumann boundary conditions. Under arbitrary perturbations, the instability or linear stability of the uniform steady states agrees with corresponding results for an ODE system involving only the reaction kinetics. We also mention that, if perturbation to the uniform steady states have large wavenumbers only, then it is possible for the zero steady state to stabilize if the diffusion parameters are large enough. Similarly, if the wavenumbers are sufficiently large, then it is possible to stabilize the positive steady states in the competition regime. Note that these stability results are valid for homogeneous steady states. We did not observe any stable heterogeneous steady states in convex domains. However, stable heterogeneous steady states may exist for some non-convex domains, as there are a number of examples of this in the literature when considering reaction–diffusion dynamics for related models on specific non-convex domains (Lou and Ni 1996; Matano and Mimura 1983). Indeed, through numerical simulations we found what appear to be heterogeneous steady states which are stable when taking the ‘dumbbell’ domain in Fig. 4, as this domain was considered for a similar purpose but with different dynamics in Matano and Mimura (1983). Meanwhile, non-convex domains with a central hole still appear to give dynamics which tend to stable homogeneous steady states.

The reaction terms are seen to determine the structure of uniform positive steady states. Indeed, such states are specified uniquely by the form of the reaction functions. Therefore, we anticipate that the reaction terms dominate for large enough time. On the other hand, advection toward resources or toward/away from other species, or dispersion throughout the domain, will occur more rapidly. Hence, advection and dispersion are responsible for transient dynamics, while asymptotics are likely dominated by reaction terms. This makes sense: For small times, movement around the domain and advection toward resources are short time, local occurrences. On the other hand, the reaction terms model the long-time fitness of the species, given their interactions and availability of ambient resources.

We have considered three biologically relevant cases when simulating the two-dimensional dynamics numerically for homogeneous domains, including competition dynamics, generalist predator-prey dynamics, and predator–prey dynamics. For each we observed transient pattern formation in many cases, with populations forming intricate and highly localized colonies, but eventually dispersing to a uniform density. Numerical simulations suggested transient dynamics with cluster formation of populations, similar to what was found in the one-dimensional case (Grindrod 1988), but all long-time dynamics that we simulated converged to uniform steady states, as our linear stability results indicate. This suggests that the model given by Eq. (5) does not exhibit stable non-uniform (or, heterogeneous) steady states when the spatial domain is convex, which is in line with simpler reaction–diffusion–advection models, e.g., (Kishimoto and Weinberger 1985). While we also considered non-convex domains with a hole in our numerical simulations, and these also appeared to give dynamics which tend to uniform steady states, it may be possible that more exotic non-convex spatial domains could give non-uniform steady states, since this may depend strongly on the form of the domain. There is some valuable information in understanding transient aggregation behaviors, particularly biologically, but the long-time dynamics appear to be dominated by the kinetics, again with the proviso that advection and diffusion parameters can modify the stability of the long-time asymptotic solutions.

While we have included numerical simulations which demonstrate interesting dynamics, note that a number of other numerical simulations were considered in order to determine the robustness of the dynamics observed. In cases with a convex domain or a domain with a central hole, all of the simulations resulted in convergence to homogeneous steady state solutions for sufficiently large time, rather than spatial patterning or non-equilibrium dynamics. This suggests that convergence to homogeneous steady states is fairly ubiquitous for this family of models when the domain is homogeneous, at least on convex and certain non-convex domains. In contrast, results on cross-diffusion mechanisms for multispecies interaction discussed earlier demonstrate that heterogeneous pattern formation is indeed possible on homogeneous domains, suggesting one measurable difference between the two classes of models.

Introducing spatially distributed resources, as we have done in Section 4, allows for non-uniform steady states, alongside more interesting transient interactions between the species. For simple spatial distributions, such as the single bump function in Fig.  5, we observe steady states that essentially recapitulate the spatially homogeneous resource dynamics by having the populations conform to the spatially distributed resources. However, for more intricate spatial structures, we observe non-trivial interactions between species, such as the abandoning of resources demonstrated in Fig. 6 that persist for asymptotically large times. We also demonstrate how this model can be used to understand migration between resource patches when the species interact during this transition. This suggests that advection along fitness gradients can induce non-trivial dispersal mechanisms, such as a separation of population centers during a migration demonstrated in Fig. 9. Finally, we considered the introduction of a hazardous region into the domain in Sect. 4.4. We observe that several phenomena are possible: extinction of one or both species, or the clustering of prey populations between hazards and predators. This later effect is an unexpected feature of the model, and we conjecture that this is due to how the predators avoid hazards around the square corners of the hazards, but the prey are able to randomly diffuse into this region.

That the incorporation of spatial heterogeneity in the fecundity can result in spatial patterning and segregation, even on convex domains, suggests that advection toward favorable or away from unfavorable regions of space can be used to strongly influence the dynamics of the populations. Interestingly, although the mechanism is a bit different, the results are akin to those concerning convergence of random motion to an ideal free distribution. In that setting, spatial forcing functions are also used to denote favorable or unfavorable regions of the domain, and under diffusion the populations find their way to favorable regions as time increases. A primary difference in our approach is that, instead of using heterogeneous forcing as an input into the reaction–diffusion system directly (as done in studies on the ideal free distribution, which results in purely local reaction–diffusion dynamics), the heterogeneity also enters into the separate advection equations in our model, and for $\varepsilon _i \ne 0$ this results in a non-local forcing toward the resource or away from the hazard. However, in both cases, one observes convergence of populations to favorable regions. As we demonstrate in numerical simulations, however, there is not uniform convergence throughout the domain, but rather there are hysteresis effects due to the initial distribution of the populations. This can result in some favorable regions being under utilized relative to others.

Both the stability results and the numerical simulations point to the fact that at small timescales, the diffusion and advection terms play a dominant role. This means that foraging and linear interactions are the primary driving mechanisms for small times, and this completely agrees with our intuition for the biological problem. Meanwhile, at larger timescales, the reaction terms become dominant, influencing both the form and the stability of steady state solutions. Indeed, for larger timescales, the overall fitness of each species plays a larger role, and this fitness includes both the ability to exploit resources as well as to survive alongside the other species (and members of the same species).

Future work could take multiple avenues. One could try and show that globally, uniform steady states are the only possible asymptotics dynamics of (5), although for two-species interactions there are some technical obstructions in the predator–prey case (see Kishimoto and Weinberger 1985 and references therein). It could be possible to consider disjoint resource subsidies, so that a predator or generalist predator would need to choose between moving toward a prey or a spatially separate subsidy. Such a scenario has been considered under the ‘Stepping-Stone’ framework (in which ODEs are defined at each separate location, rather than a continuous spatial domain); see Nevai and Gorder (2012). Furthermore, one might consider the inclusion of non-autonomous or stochastic forcing terms in order to model seasonality and other perturbations to the system. Similarly, one might consider domains with time-dependent boundaries, in order to model the effects of flooding or other natural disasters which would limit domain availability to the populations.

The results for the reaction–diffusion–advection models we consider point to the lack of pattern formation on larger timescales when there is no heterogeneous forcing present; indeed, the stability results exclude the possibility of Turing instabilities. This is in contrast to cross-diffusion type models, for which pattern formation was shown to be possible (Lou and Ni 1996). Note, however, that one might consider other reaction kinetics rather than simply quadratic interactions, which might result in Turing instability and hence pattern formation. This would be an interesting direction for future work. Additionally, one might consider a composite model, incorporating both advection toward resource gradients and cross-diffusion terms, in order to see the relative dominance of each effect on the resulting dynamics. Such a model with both nonlinear diffusion and non-local advection would likely result in a variety of the aforementioned dynamics in relevant parameter regimes, and spatial forcing would likely not, particularly if the reaction kinetics were selected in such a way that Turing instabilities are permitted.

Mathematically, it may be useful to study rotational forms of the $\mathbf {w}_i$, since for simplicity we have restricted our attention to irrotational advection by assuming that the $\mathbf {w}_i$ can be written as the gradient of a potential function. Indeed, while we have seen motion around a centrally placed barrier or obstruction, we would need to permit the $\mathbf {w}_i$ to have non-trivial rotational parts in order to obtain circular motion due to a resource or avoidance of other species in a uniform domain without obstructions. The drawback would lie in the need to consider more complicated equations for the $\mathbf {w}_i$, resulting in a system of six, rather than four, nonlinear PDEs. Aside from steady states, one could attempt to find other classes of non-uniform steady states, or even time-dependent asymptotic solution structures, such a traveling waves or quasi-steady structures which describe non-steady predator–prey pursuit and evasion dynamics. We observed none of these in our simulations, although we suspect that they exist for certain choices of the kinetic parameters and spatial domains.
